# Molecular Docking Insight into the Label-Free Fluorescence Aptasensor for Ochratoxin A Detection

**DOI:** 10.3390/molecules28124841

**Published:** 2023-06-18

**Authors:** Hua Ye, Mengyuan Wang, Xi Yu, Pengfei Ma, Ping Zhu, Jianjun Zhong, Kuo He, Yuanxin Guo

**Affiliations:** 1 School of Grain Science and Technology, Jiangsu University of Science and Technology, Zhenjiang 212100, China; 2 School of Food Science and Technology, Jiangnan University, Wuxi 214122, China; 3 College of Agriculture and Forestry Science and Technology, Hebei North University, Zhangjiakou 075000, China

**Keywords:** ochratoxin A, G-quadruplex aptamer, ThT dye, label-free, binding region

## Abstract

Ochratoxin A (OTA) is the most common mycotoxin and can be found in wheat, corn and other grain products. As OTA pollution in these grain products is gaining prominence as a global issue, the demand to develop OTA detection technology has attracted increasing attention. Recently, a variety of label-free fluorescence biosensors based on aptamer have been established. However, the binding mechanisms of some aptasensors are still unclear. Herein, a label-free fluorescent aptasensor employing Thioflavin T (ThT) as donor for OTA detection was constructed based on the G-quadruplex aptamer of the OTA aptamer itself. The key binding region of aptamer was revealed by using molecular docking technology. In the absence of the OTA target, ThT fluorescent dye binds with the OTA aptamer to form an aptamer/ThT complex, and results in the fluorescence intensity being obviously enhanced. In the presence of OTA, the OTA aptamer binds to OTA because of its high affinity and specificity to form an aptamer/OTA complex, and the ThT fluorescent dye is released from the OTA aptamer into the solution. Therefore, the fluorescence intensity is significantly decreased. Molecular docking results revealed that OTA is binding to the pocket-like structure and surrounded by the A29-T3 base pair and C4, T30, G6 and G7 of the aptamer. Meanwhile, this aptasensor shows good selectivity, sensitivity and an excellent recovery rate of the wheat flour spiked experiment.

## 1. Introduction

Ochratoxin A (OTA) is a toxic secondary metabolites produced by *Aspergillus* and *Penicillium*, that is commonly found in grain and grain products such as wheat, corn and wheat flour, as well as in fruit, wine, milk and meat products [1]. Long-term consumption of OTA-contaminated food can cause damage to the liver and kidney functions, as well as teratogenic, mutagenic and carcinogenic effects to humans with high chemical stability and thermostability [2,3,4]. It has been classified as a group 2B carcinogen by the International Agency for Research in Cancer (IARC) [5]. Due to its toxicity, many countries and organizations have established a limited standard for OTA in food and grains. The European Union (EU) and China stipulate the maximum allowable content of OTA in processed cereal and cereals products at 5.0 μg/kg (about 12.5 nmol/L), respectively [6,7]. Hence, for monitoring food safety and ensuring human health, constructing simple, fast, sensitive and reliable analytical methods for OTA detection is of great significance and urgency.

Traditionally accepted detection methods have been developed and applied for the analysis of OTA in a variety of cereals and cereals products, including wheat flour, red wine and beer. These are mainly high performance liquid chromatography (HPLC), thin layer chromatography (TLC), capillary electrophoresis analysis (CE), etc. [8,9,10]. Although these technologies possess good performance, such as highly sensitive and accurate results, they still suffer from some disadvantages, including expensive instruments, complicated operation, laborious and time consuming [11].Therefore, these methods are difficult to meet the requirements of rapid detection and on-site detection of real samples. The popular enzyme linked immunosorbent assay (ELISA), which is based on the interaction of the OTA between antibodies, is also highly sensitive in detection OTA; however, the instability of antibody and laborious, expensive preparation process bring uncertainty to the detection results. 

In recent years, aptamers are single-stranded oligonucleotide molecules selected by in vitro technology called systematic evolution of ligands by exponential enrichment (SELEX); they have drawn increasing attention to explored analytical biosensors due to their easy modification, low cost and high specificity [12,13]. As an attractive recognition element, they can be folded into a specific three-dimensional structure induced by their targets and bind with them through hydrogen bonding, electrostatic interactions and van der Waals forces, etc. [14,15,16]. Moreover, as a recognition probe, aptamers have the advantages of selectively binding to target molecules with high affinity, good specificity and wide targets recognition range. Because nucleic acid aptamers can be chemically synthesized, they have the characteristics of stable properties and low immunogenicity, and can also design corresponding complementary chains according to the base pairing principle to develop new molecular probes, which have been widely used. Up to now, various aptamer-based biosensors have been explored for the detection of OTA, such as colorimetric biosensor [17], fluorescence biosensor [18], electrochemiluminescence biosensor [19] and so on. Among these, fluorescence biosensor is a common assay that has obtained fascinating interests, due to its real-time visual observation, simplicity, rapidity and lower cost. However, they require covalent labeling of fluorophores at the ends of the aptamer molecules, which may decrease the affinity and specificity of the aptamers and increase the detection cost [20]. 

G-quadruplex is a special guanine (G)-rich nucleotide structure that can be stacked with tetrads, which is shaped of several planar guanines by hydrogen bonds [21,22]. Given such a unique property, some studies have been carried out to verify the possibility of integrating G-quadruplexes with the label free fluorescence technique for detection. The most fascinating characteristics of G-quadruplex is that it can bind with fluorescent dyes such as thioflavin T (ThT) and Hemin to enhance the fluorescence intensity [23]. ThT is a small organic molecule with very weak fluorescence intensity when it exists alone, but it can significantly enhance the fluorescence emission intensity of ThT when it is specifically combined with the G-quadruplex aptamer. ThT has good specificity, low background, low price and convenient use, so it has received extensive attention. At present, G-quadruplex is often used in combination with nucleic acid amplification or nano-signal amplification technology to improve sensitivity, which can be used for target recognition and auxiliary signal output. Due to this advantage, it can be used as a signal transduction tool to construct a set of biosensors, such as colorimetric, electrochemical and fluorescence for analysis and detection [24,25,26,27]. The currently reported OTA aptamer is a 36 bp long sequence containing 17 G base [28], and it was found to contain a Quadruplex forming G-Rich Sequence (QGRS) unit through the QGRS Mapper online tool (https://bioinformatics.ramapo.edu/QGRS/index.php, accessed on 22 February 2021) analysis. It means that the OTA aptamer is a G-quadruplex aptamer. Therefore, it is feasible to develop a label-free fluorescence aptamer biosensor by using the G-quadruplex OTA aptamer itself.

Based on the above analysis, in this work, a label-free OTA fluorescence aptasensor was established using the signal molecule ThT and the G-quadruplex OTA aptamer, and it was successfully applied to the detection of OTA in wheat flour samples. In order to better understand the binding mechanism of this biosensor, the key binding region of aptamer was revealed by using molecular docking technology. Moreover, this aptasensor provided the advantages of simple operation, good selectivity, high sensitivity and low cost, and has reliable applicability potential.

## 2. Results and Discussion

### 2.1. Scheme of the Proposed Aptasensor for OTA Detection

**Figure 1:**
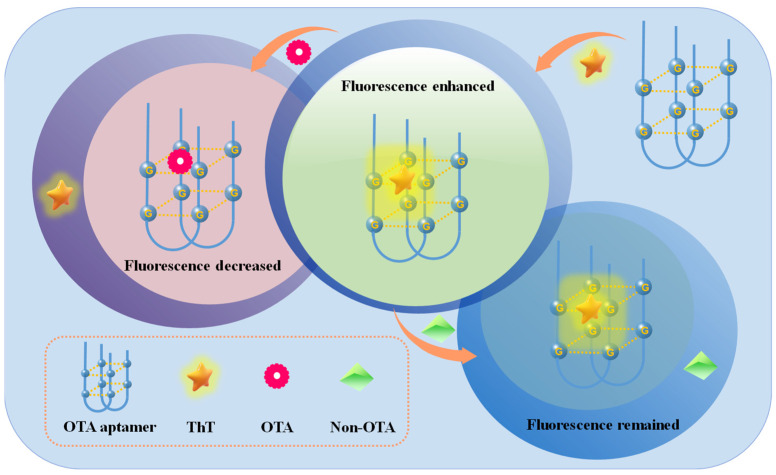
Schematic presentation of G-quadruplex-based label-free fluorescence aptasensor assay.

ThT is a water-soluble benzothifile dye with excellent chemical stability and low-fluorescence background. As mentioned above, the ThT dye is characterized by an obvious structural selectivity for G-quadruplexes, but not for other DNA forms. It can interact with G-quadruplexes through end stacking, groove binding or intercalation, which result in an enhanced fluorescence phenomenon [29]. Based on this, G-quadruplexes can be introduced as a signal indicator for various aptasensors construction to detect some targets, including heavy metal ions, antibiotics, organophosphate pesticide and other food contaminants [22]. However, the reported G-rich OTA aptamer has a G-quadruplexes structure itself which can easily achieve this design. Figure 1 represents the principle of the label-free fluorescence aptasensor for OTA detection. Due to the fact that OTA aptamer is a G-quadruplex structure itself, in the absence of the OTA toxin, the ThT fluorescent dye can bind to the OTA aptamer forming an aptamer/ThT binary complex which results in the enhanced fluorescence intensity. However, in the presence of the OTA toxin, the OTA aptamer was bound specifically with the OTA toxin due to its high affinity and specificity, thus resulting in the ThT dye release from the aptamer into the solution, and the fluorescence intensity being significantly reduced. Therefore, the detection of the OTA toxin can be realized according to the efficiency change of the fluorescence intensity.

### 2.2. Feasibility of the Proposed Aptasensor for OTA Detection

**Figure 2:**
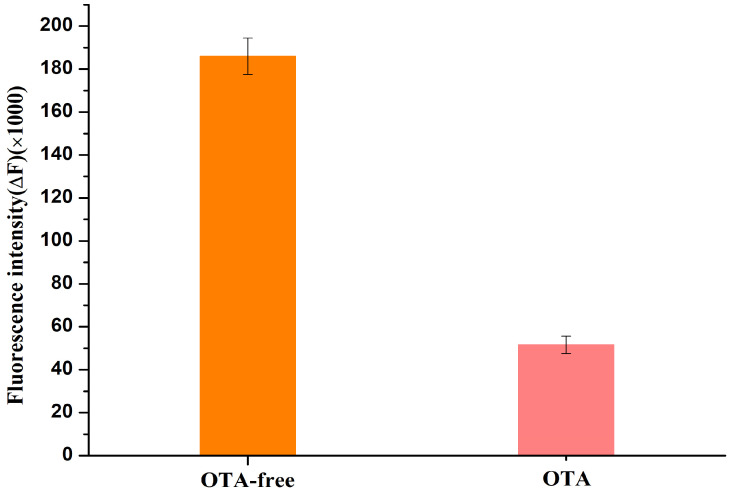
Fluorescence intensity of the aptamer/ThT mixture without OTA and with OTA (150 nM).

To evaluate the feasibility the proposed label-free fluorescence aptasensor, the fluorescence intensity of the aptamer/ThT mixture without OTA and with OTA were measured after incubating for 10 min at room temperature in the dark, and the results are shown in Figure 2. In the absence of the OTA toxin, the fluorescence intensity of the mixture was quite strong because of the binding between the OTA G-quadruplexes aptamer and ThT dye; while, with the addition of 150 nM OTA toxin, because of the high affinity of the OTA aptamer and OTA toxin, OTA toxin replaced ThT dye binding with the G-quadruplexes OTA aptamer to form a new binary complex. Therefore, the ThT dye released from the G-quadruplexes aptamer into the solution resulted in a significant decreased change in the fluorescence intensity. These experiments clearly suggest that the proposed label-free fluorescence aptasensor for OTA toxin detection is effective and feasible.

### 2.3. Molecular Docking between OTA and Its Aptamer

**Figure 3:**
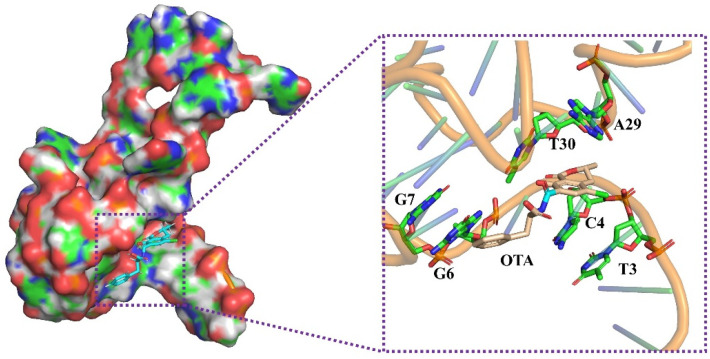
Molecular docking results between OTA and its aptamer.

Xu’s research found that the OTA toxin does not interact directly with the OTA G-quadruplex aptamer. They put forward the idea that the OTA toxin binds to the OTA aptamer at the junction region between the double helix and G-quadruplex specifically [14]. Therefore, based on their research, we further study the recognition mechanism by using molecular docking technology. The molecular docking was performed by UCSF DOCK 6.9 software (UCSF, University of California, San Francisco, CA, USA) and analyzed by the PyMoL 2.5 (Delano Scientific LLC, San Carlos, CA, USA) molecular visualization system. The result is shown in Figure 3. The OTA toxin is binding to the pocket-like structure and surrounded by six key bases, including the A29-T3 base pair and C4, T30, G6 and G7. According to Xu’s study, the A29-T3 base pair has a key contribution to their binding; this base pair directly interacts with the isocoumarin ring of the OTA toxin though π-π stacking. Significantly, C4 base is also an important base that binds with the OTA toxin with high affinity. Therefore, C4 and A29-T3 base pair are the key bases with the recognition between the OTA toxin and its aptamer, which is minorly different from Guo’s report [30]. However, Guo ‘s research indicated that C28, A29 and T30 are the three key bases of the aptamer and take part in binding to the OTA toxin. These illustrate that A29 is the most critical base and plays a crucial role in binding between the OTA toxin and its aptamer. As mentioned above, the ThT dye interacts directly with G-quadruplex to produce strong fluorescence. Therefore, the OTA toxin replaced the ThT dye and binds with the G-quadruplex OTA aptamer specifically resulting in the reduction of the fluorescence intensity.

### 2.4. Optimization of OTA Aptamer Concentration

**Figure 4:**
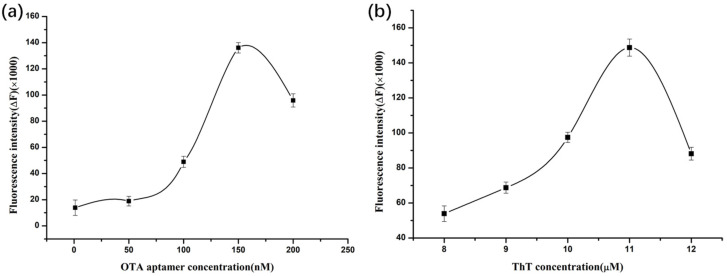
Effects of different OTA aptamer (**a**) and ThT (**b**) concentrations on fluorescence intensity.

Based on the pre-experiments, it was found that different concentrations of OTA aptamers have different effects on the fluorescence intensity of the ThT dye. Therefore, the concentration of the OTA aptamer was optimized first, and the results are shown in Figure 4a. From the figure, we can see that under the 10 μM of ThT dye solution, as the concentration of OTA aptamer increased, the fluorescence intensity of the system increased continuously. When the concentration of OTA aptamer reached to 150 nM, the fluorescence intensity reached the maximum value. However, as the OTA aptamer concentration continued to increase, the fluorescence intensity decreased. Based on the above results, under the condition of 10 μM ThT dye solution, the optimal concentration of the OTA aptamer is 150 nΜ.

### 2.5. Optimization of ThT Dye Concentration

In previous experiments, we also found that different concentrations of the ThT dye had different effects on the fluorescence intensity of the system. To make the assay more reliable, the concentrations of ThT dye were also optimized based on the optimal aptamer concentration. The results are shown in Figure 4b. Under the condition of 150 nΜ OTA aptamer, as the concentration of the ThT dye increased from 8 μM to 12 μM, the fluorescence intensity of the system increased first and then decreased sharply. When the concentration of the ThT dye solution reaches to 11 μM, the fluorescence intensity is the strongest. As the concentration of the ThT dye solution was above than 11 μM, the fluorescence intensity of the mixture decreased instead. Therefore, the optimal concentration of the ThT dye solution was 11 μΜ under 150 nΜ of OTA aptamer concentration.

### 2.6. Sensitivity Performances of the Label-Free Fluorescence Assay

**Figure 5:**
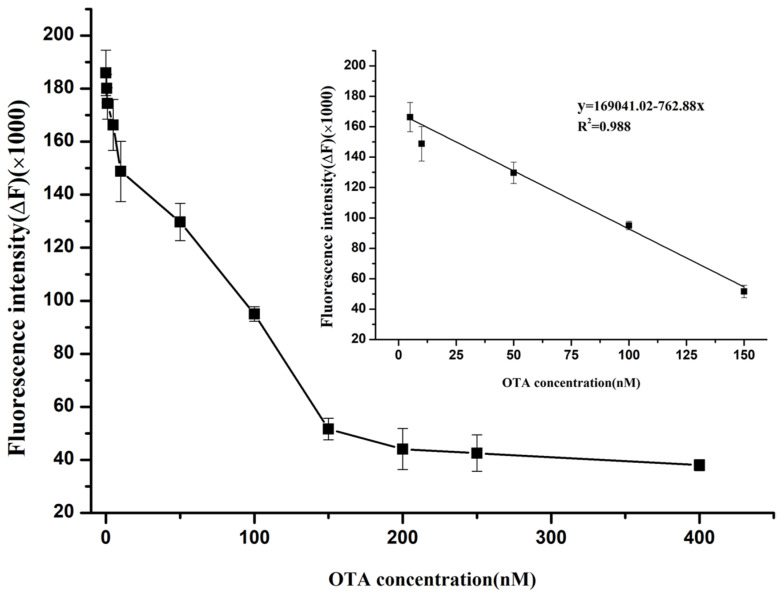
Calibration plot of changes in fluorescence intensity with various concentration of OTA.

To evaluate the sensitivity of the label-free fluorescence assay, a calibration curve was established with different OTA concentrations (0~400 nM) under the optimal conditions. Figure 5 shows that in the range of 0–400 nM OTA toxin, the fluorescence intensity decreased gradually as the concentration of the OTA toxin increased. The fluorescence intensity of the system at 483 nm has a good linear correlation to the concentration of the OTA toxin in the range of 5~150 nM. The linear regression equation of the OTA toxin was y = 169,041.02 − 762.88x (R^2^ = 0.988), and the limit of detection (LOD) (S/N = 3) of the OTA toxin was calculated as 3.42 nM, which was equivalent approximately to 1.38 μg/kg. This detection level was lower than the limited standard of the lowest OTA toxin requirement (5.0 μg/kg) set by the authorities, and meets the demand of actual detection.

To further illustrate the advantages of the proposed assay, the performance of the label-free fluorescence aptasensor was compared with other reported label-free fluorescence aptasensor methods. To make the comparison more intuitive, we have converted the units. As shown in Table S1, most of the label-free method can meet the maximum permissible concentration at 5 μg/kg (according to 12.5 nM), including our work. It is worth noting that different fluorescent donors and food samples may be produced by different LODs. Although a label-free fluorescent aptasensor based on Tb^3+^ and magnetic beads obtained a higher sensitive performance, it is poisonous to our health [31]. We also found in Table S1 that Wu et al. report a lower LOD (0.99 nM) than our work (3.42 nM) in the same fluorescent donor. This may be due to the fact that our samples are wheat flour, which is different from the liquid red wine sample. All the above results and comparisons showed that this label free fluorescence assay has high application potential in the determination of OTA, not only in red wine, but also in wheat flour and other food samples.

### 2.7. Selectivity Performances of the Label-Free Fluorescence Assay

**Figure 6:**
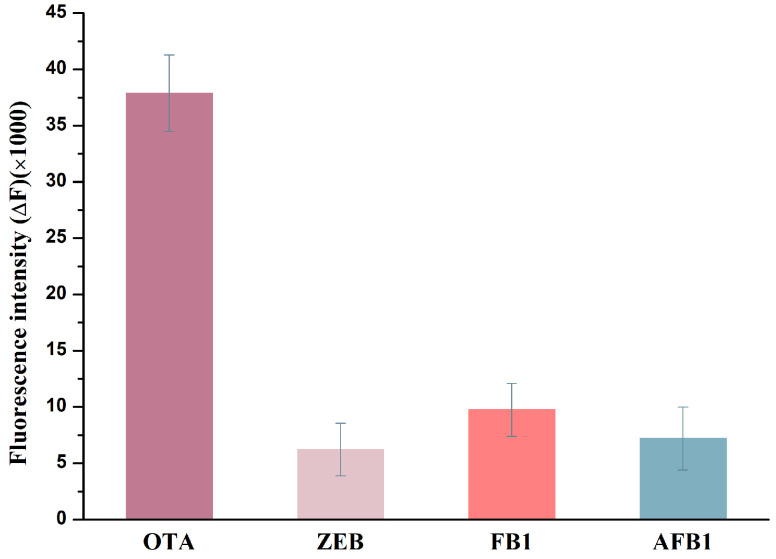
Selectivity assessment of the aptasensor for OTA.

To investigate the selectivity performances of the proposed assay for OTA toxin detection, three interfering substances including ZEN, AFB1 and FB1 were challenged. The concentration of these interfering mycotoxins (120 nM) was double the concentration of the OTA target (60 nM). As shown in Figure 6, these non-target mycotoxins interferences had almost no significant effect on the fluorescence intensity of the detection system, while in the presence of the OTA target, the fluorescence intensity induced obvious changes. These results showed that the proposed label-free fluorescence assay for OTA toxin detection possessed good specificity and provided the basis for the application in real food samples testing. However, it is worth noting that this label-free fluorescence assay needs to avoid the influence of other G-quadruplex and other interferences that can be induced by the changes in the fluorescence intensity of ThT dye.

### 2.8. Recovery Analysis in Spiked Wheat Flour Sample

To further validate the feasibility and actual application of the proposed label-free fluorescence assay, the quantitative determination of the OTA toxin in wheat flour samples was carried out to test the practicability of the assay based on the previous procedure under the optimal condition. Three different concentrations of the OTA target (5 nM, 15 nM and 100 nM) were spiked in the treated wheat flour samples, respectively. The samples without OTA were used as the control and the fluorescence intensity at 483 nm was recorded. The results as shown in Table 1, the recoveries for the OTA target in spiked treated wheat flour samples of the proposed assay were in the range of 82.89~103.2%, and the relative standard deviations (RSDs) were 2.23~6.10%. These results indicated that the proposed label-free fluorescence assay possessed good accuracy and could be applied for OTA toxin detection in wheat flour samples.

## 3. Material and Methods

### 3.1. Materials and Reagents

The OTA aptamer with a length of 36 bp utilized in this study was screened and optimized by Jorge et al., having a dissociation constant (Kd) of 200 nM and a reliable specificity [28]. The aptamer sequence is as follows: 5′-GAT CGG GTG TGG GTG GCG TAA AGG GAG CAT CGG ACA-3′, which was synthesized and purified using high-performance liquid chromatography by Sangon Bioengineering Technology and Services Co., Ltd. (Shanghai, China). Thioflavin T (ThT) fluorescent dye was purchased from Shanghai Macklin Biochemical Co., Ltd. (Shanghai, China). The biological reference standards including OTA, fumonisin B1 (FB1), aflatoxin B_1_ (AFB_1_) and zearalenone (ZEN) were purchased from Qingdao Pribolab Biological Engineering Co., Ltd. (Qingdao, China). The wheat flour products were provided by Changshen Supermarket (Zhenjiang, China). Binding buffer (BB) was prepared in the laboratory with 50 mM Tris-HCl, 5 mM KCl, 100 mM NaCl and 1 mM MgCl_2_·6H_2_O (pH 7.4). All these chemical reagents used in the experiment were analytical grade and purchased from Shanghai Macklin Biochemical Technology Co., Ltd. (Shanghai, China).

### 3.2. Instruments

SpectraMax i3x multi-mode microplate reader (Molecular Devices, San Jose, CA, USA) was used for the fluorescence intensity analysis. Eppendorf centrifuge 5427R (Eppendorf, Hamburg, Germany) was used to separate samples. The black 96-well plates were purchased from Haimen Huabo experimental equipment Factory (Nantong, China). The ultrapure water produced by Medium-E400UP ultra-pure water purifier (HHitech, Shanghai, China) was used to prepare all solutions in this experiment.

### 3.3. Optimization of OTA Aptamer Concentration

The synthesized unlabeled OTA aptamer was first dissolved in binding buffer to prepare stock solutions and then diluted to work solutions with various concentrations of 50, 100, 150, 200 nM. Then, 100 μL different concentrations of these diluted label-free OTA aptamers wasmixed thoroughly with 50 μL 10 μM of ThT fluorescent dye solution. The above mixture was incubated for 5 min at room temperature to ensure the stable binding of the OTA aptamer and ThT fluorescent dye. After incubation, the fluorescence intensity was measured on SpectraMax i3x multi-mode microplate reader with the excitation and emission wavelength of 425 nm and 483 nm, respectively. Binding buffer group was used as the control. All experiments were repeated in triplicate and carried out under dark conditions. The fluorescence intensity of the control group and experimental group was recorded as F_0_ and F_1_, respectively. The relative fluorescence ΔF (ΔF = F_1_ − F_0_) was calculated to evaluate the effect of different aptamer concentrations on the fluorescence intensity of the ThT dye solutions.

### 3.4. Optimization of ThT Dye Concentration

Based on the above label-free OTA aptamer concentration optimization results, 50 μL of different concentrations of the ThT dye solution (8, 9, 10, 11, 12 μM) was added to 100 μL of the OTA aptamer (150 nM), respectively. After shaking and incubating at room temperature for 5 min, the fluorescence intensity (F’_1_) of the solution was detected on SpectraMax i3x multi-mode microplate reader with the excitation and emission wavelength of 425 nm and 483 nm, respectively. Binding buffer group was used as a control and the fluorescence intensity was recorded as F’_0_. The relative fluorescence ΔF (ΔF = F’_1_ − F’_0_) was calculated to evaluate the effect of the ThT dye concentration on the system fluorescence intensity. Each experiment was tested in three times.

### 3.5. Label-Free Fluorescence Analysis of OTA Target

Then, 100 μL of 150 nM unlabeled OTA aptamer and 50 μL of 11 μM ThT dye solution were mixed together and vortexed for 1 min. After incubating in the dark at room temperature for 5 min, the fluorescence intensity (Fi) was recorded under the excitation and emission wavelengths of 425/483 nm, respectively. Subsequently, different concentrations (0, 0.5, 1, 5, 10, 50, 100, 150, 200, 250, 400 nM) of OTA toxin were added to the above mixture solutions, respectively. To make sure that the OTA target and OTA aptamer interacted with each other sufficiently, the mixture was incubated at room temperature in the dark for 10 min. Finally, the fluorescence intensity at 483 nm was recorded as Fii. The relative fluorescence intensity ΔF (ΔF = Fi − Fii) was calculated. A calibration curve was plotted according to the relationship between ΔF and the concentration of OTA toxin. All experiments were performed in triplicate.

### 3.6. Selectivity Analysis for OTA Detection

To evaluate the selectivity of the proposed assay for OTA toxin detection, some non-target mycotoxins such as zearalenone (ZEN), fumonisins B1 (FB1) and aflatoxin B1 (AFB1) were selected as homologous interferents. The concentration of these interfering mycotoxins was 120 nM, while OTA target concentration was 60 nM. The fluorescence intensity of the mixture was measured under the optimal operating condition as described above. The OTA group was employed as positive controls and the Binding buffer group was employed as negative controls. All experiments were performed in triplicate.

### 3.7. Determination of OTA in Wheat Flour Sample

To explore the potential application of the assay, the detection of OTA toxin in wheat flour sample was carried out. The sample test was treated based on Khan’s method, with minor changes [4]. Firstly, 5.0 g of wheat flour was added to 25 mL of 50% methanol solution, then vortexed about 20 min. After incubating for 20 min, the mixture was centrifuged at 4200 rpm for 15 min. The supernatant was collected and filtered through 0.22 μm to obtain the wheat flour sample extract. Finally, OTA toxin was spiked to prepare different target concentrations (5, 15, and 100 nM.) for analytical determination. The fluorescence intensity at 483 nm was measured on SpectraMax i3x multi-mode microplate reader under the conditions as described previously. The Binding buffer group (0 nM OTA) was employed as controls. All experiments were performed in triplicate.

### 3.8. Molecular Docking

To better understand the interaction mechanism of the OTA toxin and OTA aptamer, molecular docking was established by UCSF DOCK 6.9 in Yinfo Cloud Computing Platform (https://cloud.yinfotek.com/, accessed on 29 December 2022) and the result was analyzed by the PyMoL 2.5 (https://pymol.org/, accessed on 30 December 2022) molecular visualization system. Firstly, the 3D structure of the OTA toxin (ID:442530) was obtained from PubChem database. The three-dimensional structure of OTA aptamer was prepared based on the 6SUU structure, which was downloaded from the protein database (PDB). In order to get a better docking result, the OTA aptamer structure was optimized in several steps, including residue repairing, protonation and partial charges assignment in AMBER ff14SB force field [30]. Six key residues were selected as possible binding sites, according to Xu’s study [14]. A box enclosing the spheres was set with center of (29.999, 13.129, 20.371) and size of (47.819, 40.156, 44.459), then grids were created by Grid module for rapid score evaluation. Finally, molecular docking was conducted under semi-flexible docking and produced different orientations. Clustering analysis was performed for candidate poses and the best scored ones were output.

## 4. Conclusions

In this study, a label-free fluorescence aptasensor for OTA toxin detection was constructed and the binding mechanism between OTA toxin and its aptamer was revealed by molecular docking. The detection was established by using the change of fluorescence signal before and after the G-quadruplex OTA aptamer combined with the ThT fluorescent dye; the docking results showed that OTA toxin is binding to the pocket-like structure and surrounded by the A29-T3 base pair and C4, T30, G6 and G7 of the aptamer. The assay has good linear correlation in the range of 5~150 nM and exhibited good sensitivity with the LOD as low as 3.42 nM (about 1.38 μg/kg). In addition, this constructed label-free fluorescence aptasensor showed excellent selectivity performance for the OTA target, even in the presence of interfering mycotoxins at a two-fold higher concentration. Moreover, the recoveries for OTA in spiked wheat flour samples had good accuracy. The above results indicated that the established assay showed easy operation, time-saving, good selectivity, excellent specificity, and could be employed for the rapid detection of OTA in wheat flour and other grain food samples.

## Figures and Tables

**Table 1:** The recoveries of OTA in wheat flour samples.

Added (nM)	Found (nM)	Recovery (%)	RSD (*n* = 3) (%)
0	-	-	-
5	5.16 ± 6.10	103.2	6.10
15	14.79 ± 3.14	98.60	3.14
100	82.89 ± 2.23	82.89	2.23

## Data Availability

Not applicable.

## Supplementary Materials

The following supporting information can be downloaded at: https://www.mdpi.com/article/10.3390/molecules28124841/s1, Figure S1: Chemistry structural of Ochratoxin A; Table S1: A list of label-free fluorescence aptasensor for OTA detection. References [31,32,33,34,35] are cited in the supplementary materials.
