# Supplementary material for: Molecular Docking Insight into the Label-Free Fluorescence Aptasensor for Ochratoxin A Detection
Source: Molecules. 2023 Jun 18;28(12):4841. doi: 10.3390/molecules28124841 (PMC10304070; doi:10.3390/molecules28124841)
Supplement: Supplementary file 1 [file molecules-28-04841-s001.zip › molecules-2332515-supplementary.pdf]

Electronic Supplementary Material for

Molecular Docking Insight into the Label-Free Fluorescence Aptasensor for Ochratoxin A Detection

Hua Ye [1,*], Mengyuan Wang [1], Xi Yu [1,2], Pengfei Ma [2], Ping Zhu [1], Jianjun Zhong [1], Kuo He [3] and Yuanxin Guo [1]

[1] School of Grain Science and Technology, Jiangsu University of Science and Technology, Zhenjiang 212100, China
[2] School of Food Science and Technology, Jiangnan University, Wuxi 214122, China
[3] College of Agriculture and Forestry Science and Technology, Hebei North University, Zhangjiakou 075000, China
* Correspondence: huaye@just.edu.cn

Table of Contents

Figure S1 Chemistry structural of Ochratoxin A

Table S1. A list of label-free fluorescence aptasensor for OTA detection

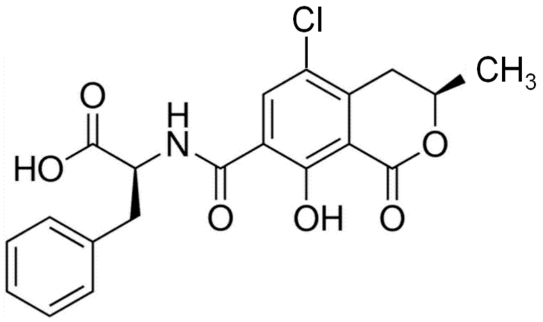

Figure S1 Chemistry structural of Ochratoxin A

Table S1. A list of label-free fluorescence aptasensor for OTA detection

Fluorescent donor	Samples	Linear Range	LOD	References
SYBR Gold	Red wine and beer	20-500 nM	16.5 nM	[1]
AIE (DSAI)	Red wine	12.38-1238 nM	4.7 nM	[2]
Thioflavin T (ThT)	Red wine	2.97-495 nM	0.99 nM	[3]
Crystal violet (CV)	Red wine	0.56-7.8 nM	0.56 nM	[4]
Tb^{3+}	Wheat	0.248-2.48 nM	49.5 pM	[5]
Thioflavin T (ThT)	Wheat flour	5-150 nM	3.42 nM	This paper

References

1. Lv, L.; Li, D.; Liu, R.; Cui, C.; Guo, Z. Label-free aptasensor for ochratoxin A detection using SYBR Gold as a probe. *Sensors and Actuators B: Chemical* **2017**, *246*, 647-652, doi:10.1016/j.snb.2017.02.143.
2. Lv, L.; Cui, C.; Xie, W.; Sun, W.; Ji, S.; Tian, J.; Guo, Z. A label-free aptasensor for turn-on fluorescent detection of ochratoxin A based on aggregation-induced emission probe. *Methods Appl Fluoresc* **2019**, *8*, 015003, doi:10.1088/2050-6120/ab4edf.
3. Wu, K.; Ma, C.; Zhao, H.; He, H.; Chen, H. Label-Free G-Quadruplex Aptamer Fluorescence Assay for Ochratoxin A Using a Thioflavin T Probe. *Toxins (Basel)* **2018**, *10*, doi:10.3390/toxins10050198.
4. Yang, C.; Dong, S.-N.; Abbas, F.; Chu, X.-L.; Fan, A.-Q.; Rhouati, A.; Mao, J.; Liu, Y. Label-free fluorescence aptasensor for ochratoxin A using crystal violet as displacement-type probe. *Chinese Journal of Analytical Chemistry* **2021**, *49*, 55-62, doi:10.1016/j.cjac.2021.07.005.
5. Zhang, J.; Zhang, X.; Yang, G.; Chen, J.; Wang, S. A signal-on fluorescent aptasensor based on Tb3+ and structure-switching aptamer for label-free detection of Ochratoxin A in wheat. *Biosens Bioelectron* **2013**, *41*, 704-709, doi:10.1016/j.bios.2012.09.053.
